# Draft Genome Sequences of Six *Vibrio* Strains Isolated from the Atlantic Intertidal Marine Sponge Ophlitaspongia papilla

**DOI:** 10.1128/MRA.01259-20

**Published:** 2021-01-07

**Authors:** Anoop Alex, Vijayakumari Pratheepa, Joana Martins, Agostinho Antunes

**Affiliations:** a CIIMAR/CIMAR, Interdisciplinary Centre of Marine and Environmental Research, University of Porto, Matosinhos, Portugal; b Department of Biology, Faculty of Sciences, University of Porto, Porto, Portugal; University of Southern California

## Abstract

We report here the genome sequences of six *Vibrio* strains isolated from an Atlantic intertidal marine sponge, Ophlitaspongia papilla. Genome mining and comparative genomics will assist in deciphering the bioactive potential of the symbiotic microbes and molecular mechanisms of sponge-microbial symbioses.

## ANNOUNCEMENT

Members of the genus *Vibrio* are a group of physiologically adapted ubiquitous and abundant bacteria inhabiting aquatic environments, including estuaries, marine coastal waters, and sediments (1–3), and several marine vertebrate/invertebrate metazoans (4–11). Furthermore, the association of members of the Harveyi clade within the genus *Vibrio* with vertebrates and invertebrates serves as a model system for understanding the molecular mechanisms of symbioses (12, 13). Despite this versatility, little is known about the diversity, distribution, and ecological relevance of *Vibrio* species living in symbiotic association with one of the basal invertebrate sponges.

A total of six *Vibrio* strains were isolated from a marine sponge, Ophlitaspongia papilla (Demospongiae), sampled from the intertidal region of the Atlantic coast (41.2308206N 8.7216926W) of Portugal. As described previously (14), approximately 1 cm^3^ sponge tissue was initially washed to remove debris and transient microbial cells, followed by grinding with sterile seawater. The tissue homogenate was serially diluted and plated onto marine agar 2216 (BD Difco, United Kingdom) medium containing amphotericin B (1 ml/100 ml) and incubated in the dark at 28°C for 3 to 4 days. Single bacterial colonies obtained after repeated streaking were inoculated into a 5-ml tube containing Difco marine broth 2216 and kept under constant shaking at 28°C. Genomic DNA was extracted from the bacterial cultures using a PureLink genomic DNA kit (Invitrogen, Carlsbad, CA, USA) according to the manufacturer’s protocol for bacterial DNA isolation. All isolates were identified as *Vibrio* by 16S rRNA gene sequencing, as described previously (15), and BLAST analysis (16).

The genomes of six *Vibrio* sp. strains, OPT10, OPT18, OPT20, OPT24, OPT41, and OPT46, were sequenced on the Illumina HiSeq 2500 sequencing system at Macrogen (Seoul, Republic of Korea). The paired-end (PE) read libraries (2 × 100 nucleotides [nt]) with an insert size of ≈350 bp were generated using a TruSeq DNA library preparation kit (Illumina) following the manufacturer’s protocols. Default parameters were used for all software unless otherwise specified. The raw data were quality filtered and adaptor trimmed using Cutadapt v1.12 (17). The processed sequence reads with a sequence quality score of >30 were assembled *de novo* using the SPAdes v3.13.1 genome assembler (18) for *Vibrio* sp. OPT10 with default parameters and the Velvet v1.2.10 (19) assembler for the remaining species with the best possible *k*-mer coverage value (*k *= 99). The genomes were annotated using the automated NCBI Prokaryotic Genome Annotation Pipeline (PGAP) (20, 21). The genome characteristic features of the sequenced strains are shown in Table 1.

**TABLE 1:** Genome information of six *Vibrio* strains isolated from a marine sponge, *Ophlitaspongia papilla*

Bacterial isolate	Genome size (bp)	No. of contigs	*N*_50_ (bp)	Total no. of genes	G+C content (%)	Final coverage (×)	GenBank accession no.	SRA accession no. (no. of reads)
OPT10^*a*^	4,995,789	69	674,397	4,425	43.5	128	JADDYL000000000	SRR12846929 (6,608,260)
OPT18	5,845,615	119	236,579	5,301	44.3	119	JADDYK000000000	SRR12846928 (6,152,344)
OPT20	5,363,590	109	348,389	4,828	44.2	116	JADDYJ000000000	SRR12846927 (6,008,632)
OPT24	4,926,672	81	416,976	4,396	43.6	129	JADDYI000000000	SRR12846926 (6,676,928)
OPT41	5,529,602	109	284,626	5,221	44.4	127	JADDYH000000000	SRR12846925 (6,528,358)
OPT46	6,243,356	910	75,765	6,393	43.8	130	JADDYG000000000	SRR12846924 (6,687,760)

a The genome was assembled using SPAdes.

The genome-wide identification of secondary metabolite gene clusters was performed with antiSMASH v5.0 (22). Seven different putative biosynthetic gene clusters (BGCs) encoding bacteriocin, betalactone, aryl polyene, ectoine, and siderophore biosynthesis and gene clusters belonging to nonribosomal peptide synthetase (100% similarity with vanchrobactin and 11% similarity with crochelin A) were detected from six *Vibrio* strains. The presence of different types of BGCs in the *Vibrio* strains warrants detailed investigation to unravel the role of metabolites in host-associated bacteria. Further analyses and comparison of genomes will be performed to search for the presence of the genomic repertoires involved in sponge-bacterium symbioses.

### Data availability.

The genome sequences of all the strains have been deposited under NCBI BioProject number PRJNA667546. The raw reads and the assemblies have been deposited in the NCBI Sequence Read Archive (SRA) and in GenBank under the accession numbers given in Table 1.
